# Characterization of Hyaluronan Localization in the Developing Mammary Gland and Mammary Tumors

**DOI:** 10.1007/s10911-023-09528-y

**Published:** 2023-01-27

**Authors:** Patrice M. Witschen, Alexis K. Elfstrum, Andrew C. Nelson, Kathryn L. Schwertfeger

**Affiliations:** 1 Comparative and Molecular Biosciences Graduate Program, University of Minnesota, Minneapolis, MN USA; 2 Microbiology, Immunology and Cancer Biology Graduate Program, University of Minnesota, Minneapolis, MN USA; 3 Department of Laboratory Medicine and Pathology, University of Minnesota, Minneapolis, MN USA; 4 Masonic Cancer Center, University of Minnesota, Minneapolis, MN USA; 5 Center for Immunology, University of Minnesota, Minneapolis, MN USA

**Keywords:** Mammary gland, Breast cancer, Hyaluronan, Macrophage, Fibroblast

## Abstract

The extracellular matrix (ECM) is biochemically and biomechanically important for the structure and function of the mammary gland, which undergoes vast structural changes throughout pubertal and reproductive development. Although hyaluronan (HA) is a ubiquitous glycosaminoglycan (GAG) of the mammary gland ECM, extensive characterization of HA deposition in the mammary gland is lacking. Understanding physiologic HA metabolism is critical as this tightly controlled system is often hijacked in cancer. In the current studies, we characterize HA regulation throughout mammary gland development to better understand subsequent dysregulation of HA in mammary tumors. Using immunofluorescence (IF) imaging, we demonstrate that organized HA-rich septa exist in the mammary gland stroma throughout puberty, pregnancy, and involution. Furthermore, we find heterogeneous HA deposition within two murine models of breast cancer. Using cell specific isolation techniques, we characterize expression of genes associated with HA binding, synthesis, and degradation within EpCAM + epithelial cells, CD90.2 + fibroblasts, and F4/80 + macrophages isolated from mammary glands and tumors. Most notably, we identify elevated levels of the hyaluronidases *Hyal1* and *Hyal2* in tumor-association macrophages (TAMs), suggesting a role for TAM-mediated turnover of HA in the tumor microenvironment (TME). Gene expression is supported functionally by in vitro experiments in which macrophages treated with tumor-cell conditioned media exhibit increased hyaluronidase activity. These findings link TAMs to the direct degradation of HA within the TME of mammary tumors, which has negative implications for patient survival.

**Supplementary Information:**

The online version contains supplementary material available at 10.1007/s10911-023-09528-y.

## Introduction

Hyaluronan (HA) is a ubiquitous glycosaminoglycan (GAG) of the extracellular matrix (ECM), consisting of alternating disaccharides that form a linear, unbranched chain [1]. Despite its simple structure, HA is vital for all stages of life, from ovum fertilization [2] and development of embryonic tissues [3] to inflammation and wound healing [4]. Existing at molecular weights of 10^6^-10^7^ Da under physiologic conditions, this negatively charged, high-molecular weight (HMW) molecule primarily maintains hydration of tissues while also providing structural support [1], [3]. HMW HA is synthesized at the plasma membrane by hyaluronan synthases (HAS) 1–3, where it is released into the stroma and bound by surrounding cells via surface receptors such as cluster of differentiation 44 (CD44), receptor for HA-mediated motility (RHAMM), and lymphatic vessel endothelial receptor 1 (LYVE1) [5]. Remarkably, the average 70-kg human contains 15 g of HA, 1/3 of which is turned over daily [6]. Physiologic HA turnover is mediated by hyaluronidases (HYAL), where it is cleaved at the cell surface by HYAL2, internalized, and further degraded by HYAL1 in lysosomes [7], [8]. HA homeostasis in normal tissues requires a well-balanced, tightly controlled system; however, deregulation of HA synthesis and degradation has been associated with cancer [9], [10].

Breast cancer remains the second-leading cause of cancer-related death among women in the United States. Because cancer cells make up a small fraction (~ 21%) of the tumor mass [11], [12], it is essential to define other components of the tumor microenvironment (TME). Namely, ECM density/stiffness has been strongly associated with breast cancer development, resulting in routine mammogram screenings for women [13], [14], thus supporting a role for ECM deposition and tumorigenesis. Therefore, it is important to identify key cell types that contribute to ECM regulation. For instance, increased tumor-associated macrophages (TAMs) [15], [16] and HA accumulation [17] have been associated with decreased overall survival in breast cancer patients, yet evidence defining this relationship is lacking. While there is a growing body of correlative evidence linking TAMs with regulation of ECM [11], [13–17], the causal relationship between stromal/tumor cells and ECM manipulation in breast cancer development is poorly understood. In order to understand how this system is altered in breast cancer progression, it is critical to understand physiologic HA metabolism.

ECM is both biochemically and biomechanically important throughout mammary gland development [18–22], which undergoes vast structural changes throughout pubertal and reproductive development [18]. At birth, the mammary gland consists of a rudimentary ductal system that is relatively quiescent. With the onset of puberty (approximately 4 weeks in mice), dramatic structural changes occur in response to estrogen and growth hormone (GH), leading to ductal elongation and branching throughout the mammary fat pad [23], [24]. Following hormone activation, this choreographed chain-of-events is largely executed by tissue resident cells such as fibroblasts and macrophages. For example, ECM remodeling is vital for estrogen-induced ductal elongation in which fibroblasts degrade the ECM via protease production (primarily MMPs) and deposit a collagen scaffold [25], [26]. Similarly, macrophages play a role in collagen production (either directly or indirectly) as their ablation led to a dramatic reduction in collagen deposition and terminal end bud (TEB) formation [27], [28]. During pregnancy and lactation, a surge in progesterone [29] and prolactin [30] induce epithelial proliferation (alveologenesis) to support milk secretion. Finally, upon lack of milk demand, epithelial apoptosis (involution) occurs, resulting in significant remodeling of the ECM [19], [20] and returning the gland to its pre-pregnant state.

Despite limited research, a few key papers have highlighted the relevance of HA in the developing mammary gland. In 2015, Tolg and colleagues [31] isolated varying sizes of HA from all stages of the remodeling mammary gland. They found that the majority of HA within the mammary gland was > 300 kDa, however, LMW HA fragments (7–21 kDa) were highest during pregnancy. Moreover, Tolg et al. demonstrated that LMW HA fragments (averaging 10 kDa) enhanced epithelial branching in the presence of epidermal growth factor (EGF) using a 3D model of epithelial morphogenesis *in vitro.* Furthermore, studies from our lab have recently defined a role for macrophages and ECM homeostasis. Specifically, in vivo depletion of resident macrophages via pexidartinib (CSF1R, c-Kit and Flt3 inhibitor) revealed a dramatic increase in HA deposition within mammary gland stroma, emphasizing a role for macrophages and HA turnover in the mammary glands of nulliparous mice [32]. Apart from these key findings, HA metabolism has not been investigated in the normal mammary gland literature. Therefore, further work is needed to better understand HA regulation in mammary gland development and subsequent dysregulation in mammary tumorigenesis.

In the current studies, we aim to characterize HA deposition within developing mammary glands and mammary tumors. Using immunofluorescence (IF) imaging, we demonstrate that organized HA-rich septa exist throughout the pubertal, pregnant, and involuting mammary glands. In contrast, we find heterogeneous HA deposition within two murine models of breast cancer (4T1 and HC11/R1-LM). Using cell specific isolation techniques, we characterize gene expression of HA machinery within EpCAM + epithelial cells, CD90.2 + fibroblasts, and F4/80 + macrophages derived from mammary glands and tumors. Most notably, we identify elevated levels of *Hyal1* and *Hyal2* in tumor-association macrophages (TAMs), suggesting a role for TAM-mediated turnover of HA in the TME. Gene expression is supported functionally through a series of in vitro experiments in which macrophages treated with tumor-cell conditioned media exhibit increased hyaluronidase activity. These findings suggest a role for TAM-mediated turnover of HA in the TME, which has negative implications for patient survival [17], [33].

## Materials and Methods

### Cell Culture

4T1 cells were obtained from Dr. Thomas Griffith, University of Minnesota, Minneapolis, MN and J774 cells were obtained from ATCC. Both cell lines were cultured per ATCC recommendations. HC11/R1 cells were obtained from Dr. Jeffrey Rosen, Baylor College of Medicine, Houston, TX, and maintained as described previously described [34]. HC11/R1-LM cells were generated to create a cell line with enhanced take rate and metastatic propensity in vivo as previously described [35], [36]. Mouse bone marrow derived macrophages (BMDMs) were isolated and maintained as previously described [37].

### Mice

BALB/cAnNHsd mice were purchased from Envigo at either 5- (pubertal) or 10-(adult) weeks old for mammary gland development studies or 6–8 weeks old for tumor transplantations. All experiments were performed using female mice housed in specific pathogen-free facilities. All animal care and procedures were approved by the Institutional Animal Care and Use Committee of the University of Minnesota and were in accordance with the procedures detailed in the Guide for the Care and Use of Laboratory Animals [38].

### In vitro stimulation of J774 macrophages with conditioned media

HC11/R1-LM cells were serum starved then stimulated with 30 nM B/B homodimerizer for 12 h, following which media was collected. 4T1 cells were serum starved and media was collected. Next, J774 cells were plated at 2 × 10^6^ cells/well of a 6-well plate. The next day, cells were starved for 4 h at 37 °C in serum free DMEM, following which fresh DMEM, HC11/R1-LM, or 4T1 conditioned media was added for 2 h. Cells were lysed in RIPA buffer containing protease inhibitors. Hyaluronidase protein was measured with a hyaluronidase ELISA (LS-Bio #LS-F9648-1) and hyaluronidase activity was measured with a hyaluronidase activity assay (Echelon #K-600).

### Mammary Gland and Tumor Collection

BALB/c mammary gland harvest: Estrous staging was confirmed using crystal-violet staining to identify cell morphology following vaginal lavage as previously described [39]. For pregnancy samples, female mice were humanely euthanized on days 8, 12, or 18 post conception (vaginal plug formation). For involution samples, pup numbers were normalized to 6–8 pups per dam. After 10–13 days of lactation, pups were removed to initiate involution (Day 0). The fourth inguinal mammary fat pads were excised for histologic analysis or Miltenyi Bead Isolation.

BALB/c mice tumor induction: 1 × 10^4^ 4T1 cells or 5 × 10^4^ HC11/R1-LM cells [35] were resuspended in 50% Matrigel/PBS solution and orthotopically injected into the fourth mammary fat pad of mice. All mice harboring HC11/R1 or HC11/R1-LM tumors received 1 mg/kg B/B homodimerizer (Clontech), intraperitoneally, twice weekly. Once palpable, tumors were measured using calipers every other day to determine growth rate and total tumor volume. Animals were humanely euthanized using CO_2_ once tumors reached endpoint (1.5-2 cm^3^). Tumors were excised for histologic analysis or Miltenyi Bead Isolation.

### Cell-Specific Isolation from Tissues

Harvested tumors or mammary glands (third and fourth mammary glands were pooled from 3 to 4 mice per sample) were minced and digested in 1 mg/mL Collagenase D (Roche) containing 15 µg/mL DNaseI (Sigma-Aldrich) at 37° C with vigorous shaking for 45 min. Following digestion, tissues were further homogenized through a 70 μm cell strainer and pelleted by centrifugation at 500x*g*. Red blood cells were lysed in ammonium-chloride-potassium (ACK) buffer (150 mM ammonium chloride, 10 mM potassium chloride, 0.1 mM sodium EDTA, pH 7.4) and resuspended in magnetic-activated cell sorting (MACS®) buffer (0.5% BSA and 2mM EDTA in PBS). Macrophages were isolated using anti-F4/80 microbeads mouse kit via positive selection (Miltenyi Biotec #130-110-443). CD45-positive cells were depleted using the anti-CD45 microbeads (Miltenyi Biotec #130-052-301) mouse kit. Next, EpCAM + cells followed by CD90.2 + fibroblasts were isolated via positive selection using the anti-CD326 microbeads (Miltenyi Biotec #130-105-958) and anti-CD90.2 microbeads (Miltenyi Biotec #130-121-278) mouse kits, respectively. Upon isolation, each cell type was further enriched by passing samples through a second MACs LS column (Miltenyi Biotec #130-042-401). Cell pellets were either lysed in TriPure trizol (Roche) for quantitative RT-PCR or resuspended in FACs buffer for flow cytometry.

### Quantitative RT-PCR

RNA was extracted from cells using TriPure trizol (Roche) and quantified using UV spectroscopy. cDNA was synthesized using the qScript cDNA synthesis kit (Quanta Biosciences) per manufacturer’s instructions. qRT-PCR was performed using PerfeCTa SYBR Green (Quanta Biosciences) and the Bio-Rad iQ5 system. Ct values were normalized to cyclophilin B (CYBP) and the 2 − ΔΔCt method was used to determine relative quantification of gene expression. Normalization to the geometric mean of the EpCAM samples was performed. Primer sequences (5’ – 3’): ***Has1***: Fwd – CAG AGC CTC TTC GCT TAC CT, Rev- TAG GCT GAG ATG GTG AGT GC; ***Has2***: Fwd – TGT GAG AGG TTT CTA TGT GTC CT, Rev- ACC GTA CAG TCC AAA TGA GAA GT; ***Has3***: Fwd – CCT ATG AAT CAG TGG TCA CAG GTT T, Rev-TGC GGC CAC GGT AGA AAA; ***Hyal1***: Fwd – TGC TCA GAA AGT TTG GAG AAT GAA G, Rev- AAA GTC AGG AAG AGA GTA GAG ATG C; ***Hyal2***: Fwd – TCT TCA CGC GTC CCA CAT AC, Rev- CAC TCT CAC CGA TGG TAG AGA TAA G; ***Cd44***: Fwd – TCT GCC ATC TAG CAC TAA GAG C, Rev- GGG AAG AGA GTC CCA TTT TCC A; ***Rhamm***: Fwd – CCT TGC TTG CTT CGG CTA AAA, Rev- CTG CTG CAT TGA GCT TTG CTT CT; ***Lyve1***: Fwd –TTC CTC GCC TCT ATT TGG AC, Rev- ACG GGG TAA AAT GTG GTA AC.

### Flow Cytometry

Experimental samples and single-stained controls were resuspended in 100 µL antibody master mix (FcR Block CD16/CD32 Monoclonal Antibody at 1:100, ThermoFisher 14-0161-82; Invitrogen™ eBioscience™ Fixable Viability Dye eFluor™ 780 at 1:1000; PE anti-mouse F4/80 antibody at 1:50, Biolegend #123,109; AF488 anti-mouse CD326 antibody at 1:100, Biolegend #118,210; APC/Cy7 anti-mouse CD90.2 antibody at 1:100, Biolegend #105,328) while unstained controls were incubated in FACS Buffer (2% FBS and 1 mM EDTA in PBS) for 30 min on ice protected from light. Following antibody staining, samples were fixed in 2% paraformaldehyde (PFA) for 30 min on ice. Cells were washed and resuspended in 300 µL FACS buffer. Flow cytometry was performed on a BD LSR Fortessa X-20 and data was analyzed via FlowJo.

### Immunofluorescence

Tumors and mammary glands were fixed in 4% paraformaldehyde and paraffin embedded. When appropriate, control sections were treated with hyaluronidase (16 U/mL) for 30 min in a humidity chamber at 37 °C. Sections were then stained with either hematoxylin and eosin (H&E) or biotinylated hyaluronan binding-protein (HABP, MilliporeSigma #385,911 at 1:100) for 1 h at room temperature. Sections were incubated in Streptavidin AF 488 conjugate (at 1:500, FisherScientific #S11223) secondary antibodies for 1 h at room temperature in a humidity chamber: Tissues were mounted with a coverslip using ProLong Gold Antifade DAPI (Invitrogen, #P36931).

### Localization of HA in paraffin-embedded tissues

In order to localize HA in paraffin-embedded tissues, we first established a staining protocol that relies on the specificity of HA binding protein (HABP) for HA [40]. The experimental design is outlined in Figure S1, where HA is first digested on control slides using hyaluronidase [8]. Serial control and experimental slides are then exposed to the same experimental protocol utilizing biotinylated HABP and a secondary streptavidin fluorophore. HA staining is then visualized under a fluorescent microscope. By comparing experimental samples with hyaluronidase-treated controls (Figure S1), we can effectively localize HA in developing mammary glands and mammary tumors.

### Microscope Imaging

Immunofluorescence and H&E images were acquired on Leica DM6000B and DM5500B microscopes, respectively, using 10x, 20x or 40x objectives. Images were acquired using a Leica DFC310 FX camera and LAS V3.8 software.

### Statistical Analysis

Statistical analysis was performed using Student’s unpaired, two-tailed *t*-test. Comparisons between multiple groups was performed using one-way ANOVA with Dunnett’s multiple comparisons test. Error bars represent standard error of the mean (SEM).

## RESULTS

### Organized hyaluronan-rich septa are localized throughout the developing mammary gland

**Fig. 1:**
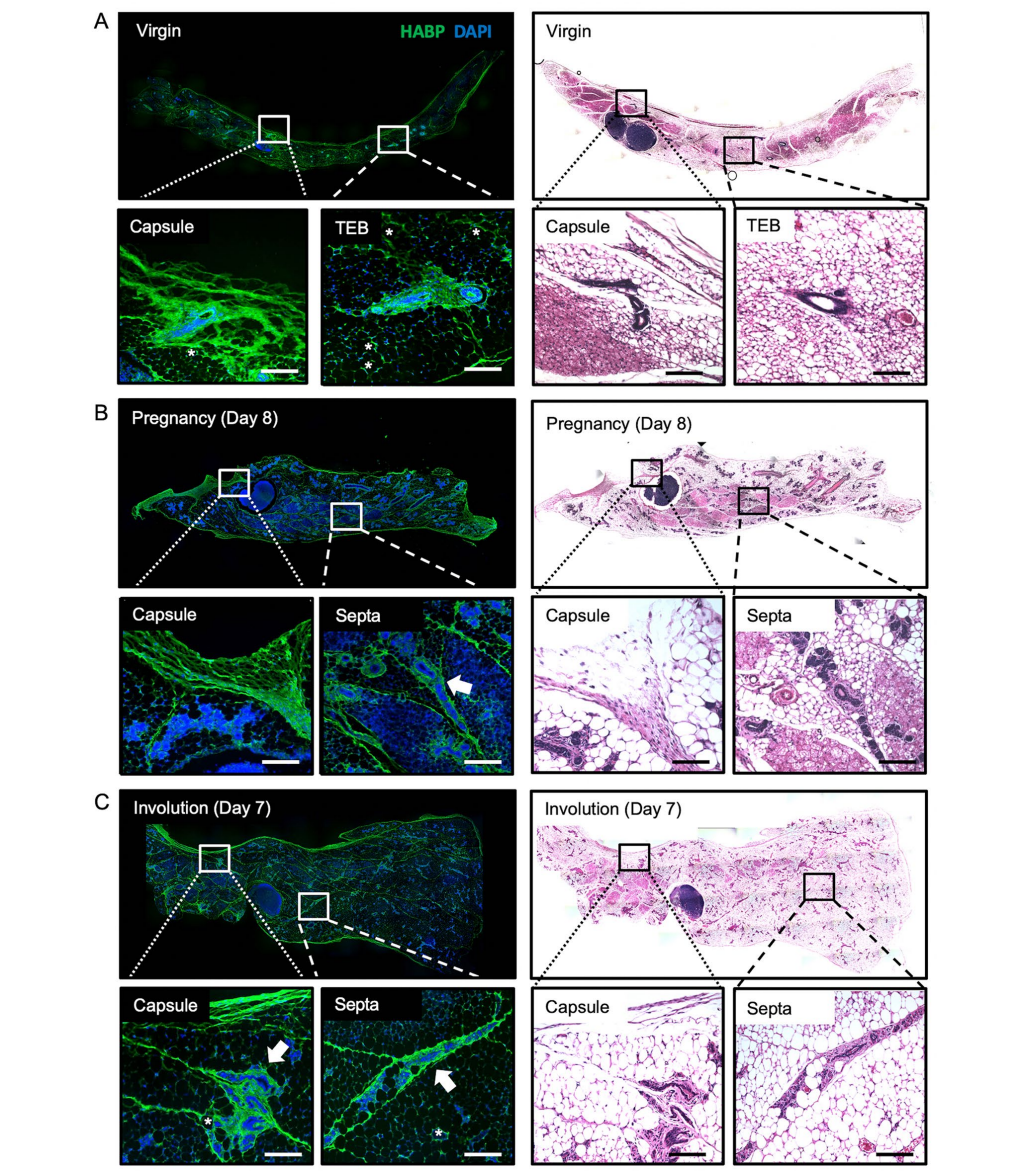
**Organized hyaluronan-rich septa are deposited throughout the developing mammary gland.** Immunofluorescence microscopy for hyaluronic acid binding protein (HABP; green) and DAPI nuclear stain alongside serial hematoxylin and eosin- stained images identifying key hyaluronan (HA) structures within mammary glands of virgin/nulliparous (**A**), pregnant (**B**), and involuting (**C**) BALB/c mice. HA extends throughout the mammary fat pad (IF images), dividing the gland into distinct lobules and encasing it in a fibrous capsule (inserts). Arrows highlight epithelial buds enveloped by HA-rich septa. Asterisks highlight adipocytes with a prominent pericellular HA coat. Whole-gland images were acquired on Leica DM6000B (IF) and DM5500B (H&E) microscopes at 100× magnification and stitched together via the LAS V3.8 software. Inserts were acquired on Leica DM6000B (IF) and DM5500B (H&E) microscopes at 200× magnification. Scale bars represent 100 µM

**Fig. 2:**
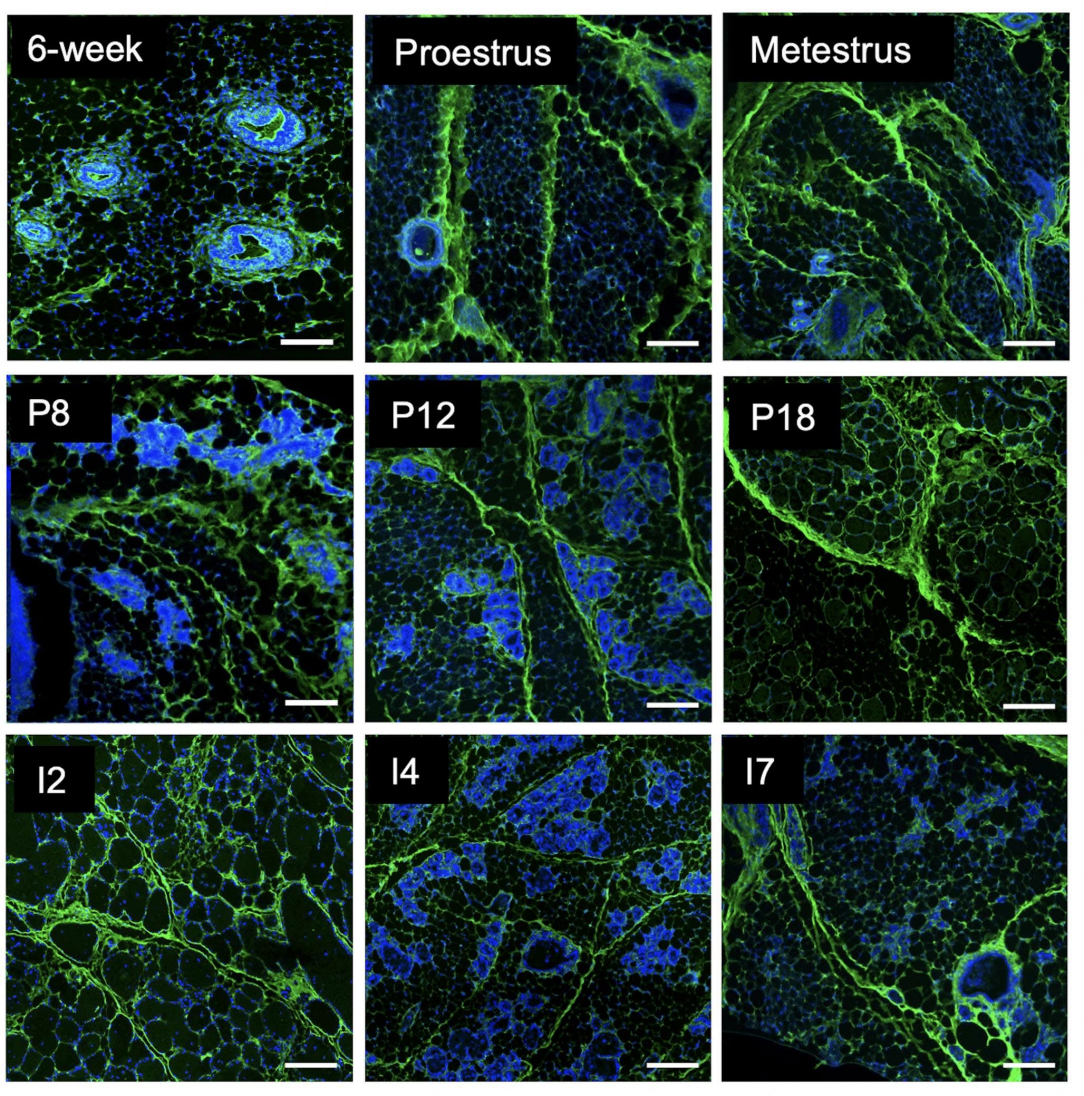
**Hyaluronan-rich septa are largely unchanged throughout mammary gland development.** Immunofluorescence microscopy for hyaluronic acid binding protein (HABP; green) and DAPI nuclear stain identifying hyaluronan (HA) septa within the mammary glands derived from nulliparous (6-week, proestrus, metestrus), pregnant (P days 8, 12, and 18), and involuting (I days 2, 4, and 7) BALB/c mice. Epithelial ducts are often found along HA-rich septa, nestled within this dense matrix. This “string-of-pearl” effect was particularly evident in images P8 and P12. Images were acquired on the Leica DM6000B microscope at 200× magnification. Scale bars represent 100 µM

To define HA localization within the mammary gland, the fourth inguinal mammary glands were obtained from nulliparous mice and during pregnancy and involution and HA was assessed using IF as demonstrated in Figure S1. Serial H&E images were used to confirm developmental status and guide the assessment of IF images. As shown in Fig. 1 A, mammary glands from pubertal mice are associated with a simple, ductal network that extends throughout the mammary fat pad, ending in terminal end buds (TEB, highlighted via insert). By day 8 of pregnancy (Fig. 1B), the mammary epithelia have appropriately undergone significant proliferation (alveologenesis), which have regressed by day 7 of involution (Fig. 1 C). Interestingly, HA extends throughout the mammary fat pad (evidenced by the IF images) and localized to distinct septa, dividing the gland into distinct lobules and encasing it in a fibrous capsule. These ECM-enriched septa likely also contain collagen and other matrices. In previously published work from our lab, trichrome staining of the mammary gland from nulliparous mice highlights collagen-containing ECM in mammary gland septa [32]. Therefore, these septa are referred to as “HA-rich” to acknowledge that HA represents a component of this matrix. Notably, epithelial buds are frequently found enveloped by an HA-rich matrix along visible HA-rich septa (highlighted by arrows in Fig. 1B,C), while individual adipocytes are often surrounded by a prominent pericellular HA coat (highlighted by * in Fig. 1 A). Because we have previously described the mammary gland capsule, which is enriched in HA [32], we aimed to further characterize HA-rich septa in Fig. 2 by imaging mammary glands during key stages of puberty, pregnancy, and involution. Interestingly, epithelial ducts are often found along HA-rich septa, nestled within this dense matrix. This “string-of-pearl” effect was particularly evident during early and mid-pregnancy (P8 and P12, respectively). Overall, the organization, distribution, and thickness of HA-rich septa were consistent across stages. Because organized HA structures were prominent throughout each stage of development, HA likely provides essential structural support to the adipose fat pad and mammary gland.

### Contributions to HA synthesis and turnover by cell type in the developing mammary gland

It is well-established that ECM is both biomechanically and biochemically important for various stages of mammary gland development, including ductal elongation/branching [18–22]. While much of this work recognizes matrices such as collagen and laminin [19], [23], [41], our findings suggest that HA also represents a ubiquitous matrix component within the developing mammary gland. Due to the complex signaling networks present during development, in vitro investigation of HA machinery would be challenging. Therefore, we opted for an ex vivo approach to investigate physiologic HA metabolism in key cell types derived from the pubertal (5-week) and adult (10 week) mammary glands of BALB/c mice. Specifically, EpCAM+, CD45-/CD90.2+, and F4/80 + Miltenyi Bead Isolation kits (outlined in Figure S2A) were used to isolate epithelial cells, fibroblasts, and macrophages, respectively. Flow cytometry was used to confirm both cell-specific enrichment and viability (Figure S2B, C).

**Fig. 3:**
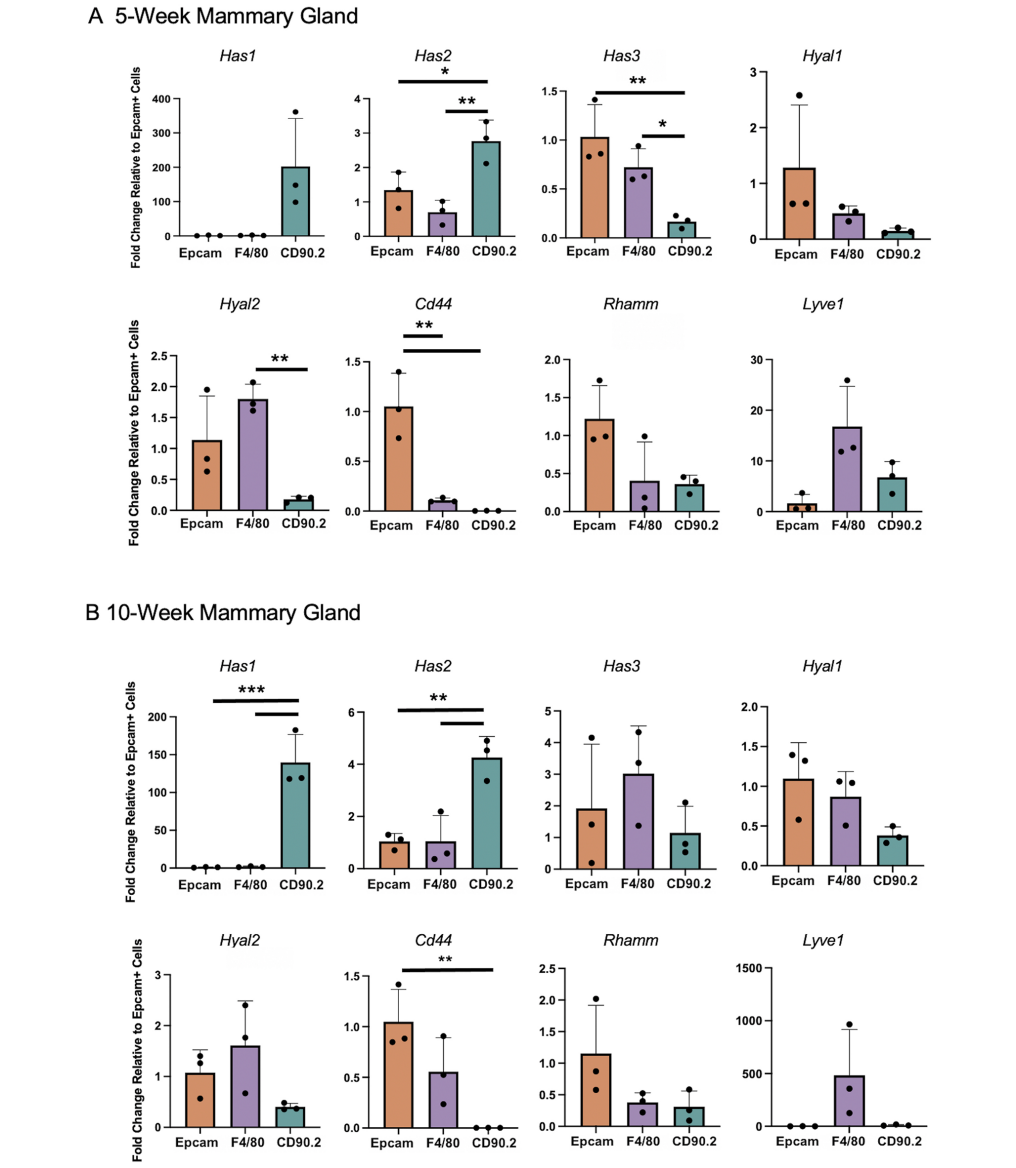
**Contributions to HA synthesis and turnover by cell type in the developing mammary gland.** EpCAM+, F4/80+, and CD45-/CD90.2 + cells were isolated from the mammary glands of (**A**) 5- (n = 3) and (**B**) 10- (n = 3) week old female BALB/c mice using the Miltenyi Bead Isolation kit. Gene expression by qRT-PCR was analyzed utilizing a panel of HA-related genes: hyaluronan synthesis (*Has1-3*), hyaluronidases (*Hyal1,2*), and hyaluronan receptors (*Cd44, Rhamm, Lyve1*). Third and fourth mammary glands were pooled from 3–4 mice per sample (n). Error bars represent standard error of the mean. *P* values * *p* < 0.05; ** *p* < 0.01; *** *p* < 0.001

To investigate expression levels of genes associated with HA machinery in the mammary gland during ductal elongation and in established mammary glands, cell-specific gene expression was analyzed within 5- (Fig. 3 A) and 10-week (Fig. 3B) cohorts utilizing primers for a panel of HA-related genes: HA synthesis (*Has1-3*), HA fragmentation (*Hyal1,2*), and HA receptors (*Cd44, Rhamm, Lyve1*). As shown in Fig. 3, *Cd44* was elevated in EpCAM + cells derived from both the 5- and 10- week-old mammary glands when compared to CD45-/CD90.2 + cells. Additionally, *Hyal2* was elevated in the F4/80 + cells derived from the 5-week old mammary gland when compared to CD45-/CD90.2 + cells, supporting our previous findings [32] in which macrophages play a significant role in HA turnover in the mammary gland. Finally, CD45-/CD90.2 + cells exhibited elevated expression levels of *Has1* and *Has2* at the 10-week time points when compared with other cell types. These data are consistent with other published studies that have demonstrated the ability of fibroblasts to synthesize HA [22], [41], [42]. Interestingly, CD45-/CD90.2 + cells specifically expressed *Has1* whereas F4/80 + and EpCAM + cells expressed elevated levels of *Has3* at the 5-week timepoint. In summary, these data are consistent with the hypothesis that all three cell types contribute to HA deposition, while macrophages may contribute to HA degradation in mammary glands at the 5-week timepoint.

### Hyaluronan is heterogeneously deposited throughout mammary tumors

**Fig. 4:**
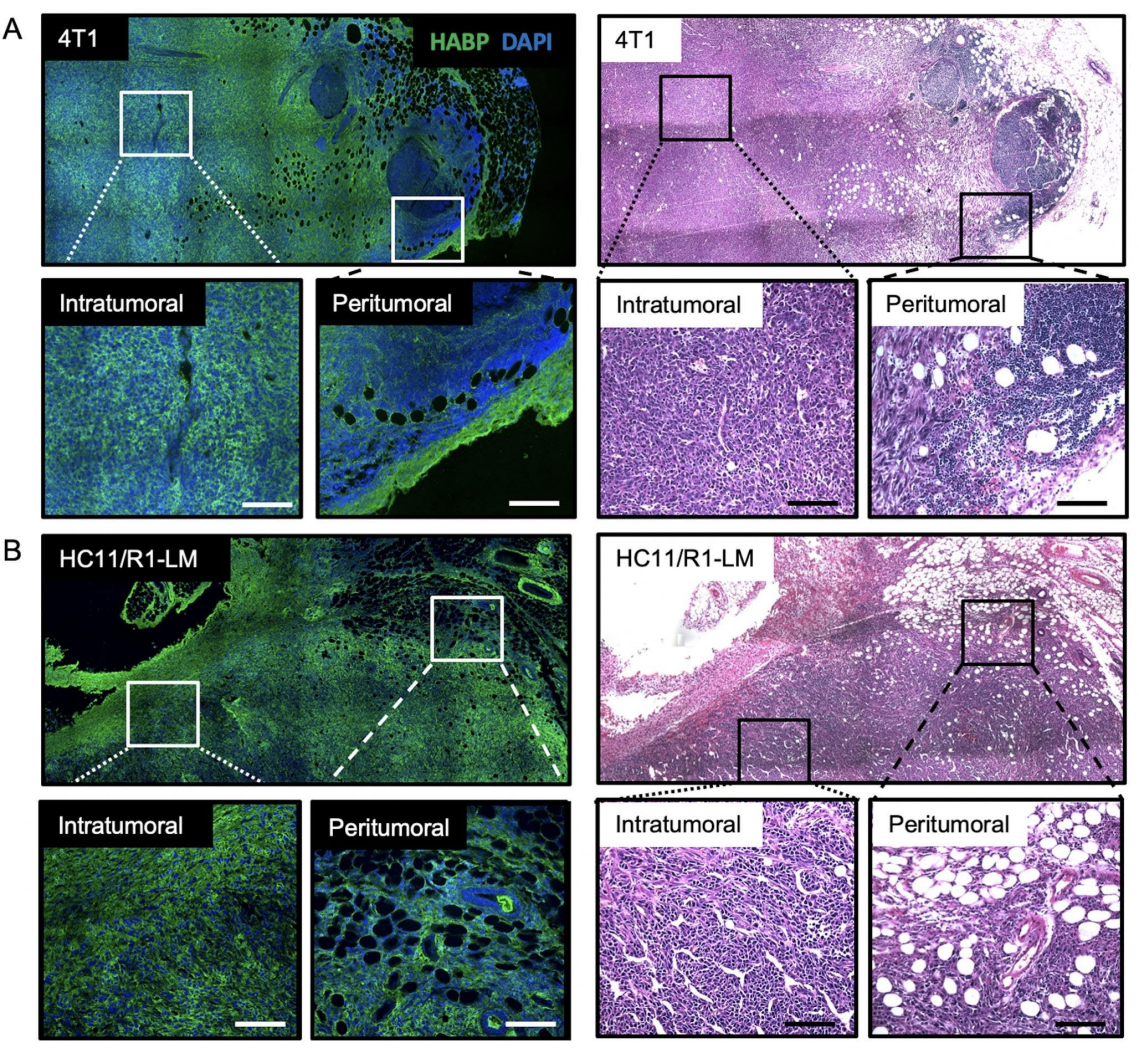
**Hyaluronan is heterogeneously deposited throughout mammary tumors.** Immunofluorescence microscopy for hyaluronic acid binding protein (HABP; green) and DAPI nuclear stain alongside hematoxylin and eosin (H&E) - stained images identifying hyaluronan (HA) deposition within three murine models of breast cancer: (**A**) 4T1 and (**B**) HC11/R1-LM. H&E images of 4T1 and HC11/R1-LM tumors highlight the highly cellular tumor adjacent to the normal, adipocyte-rich mammary gland. Inserts identify intratumoral and peritumoral regions within each tumor. Whole tumor images were acquired on Leica DM6000B (IF) and DM5500B (H&E) microscopes at 200× and 100× magnification, respectively, and stitched together via the LAS V3.8 software. Inserts were acquired on Leica DM6000B (IF) and DM5500B (H&E) microscopes at 20× magnification. Scale bars represent 100 µM

Increased HA deposition has been linked to poor patient survival in breast carcinomas [17], [43], among others [10], [43], [44]. However, HA deposition/localization has not been characterized in the 4T1 or HC11/R1-LM mammary tumors. Therefore, we utilized IF staining to evaluate HA deposition mapped to serial H&E sections within the two murine models of breast cancer. H&E images of 4T1 (Fig. 4 A) and HC11/R1-LM (Fig. 4B) tumors highlight the highly cellular tumor infiltrating into the normal adipose tissue of the surrounding mammary gland. Both peritumoral and intratumoral regions show heterogeneous HA deposition, which has previously been associated with poorly differentiated breast tumors [17]. In contrast to the organized HA-rich septa in normal mammary glands, HA accumulates in a diffuse, unstructured pattern throughout tumors derived from two murine models of breast cancer.

### Contributions to HA synthesis and turnover by cell type in mammary tumors

Because cancer cells make up a small fraction (~ 21–25%) of the tumor mass including in mouse mammary tumor models [11], [12], it is essential to define other components of the TME. Thus, we next characterized HA regulation in cells derived from mammary tumors. To address this, EpCAM+, F4/80+, and CD45-/CD90.2 + cells were isolated from two murine models of breast cancer (4T1 and HC11/R1-LM) using the Miltenyi Bead Isolation kit (outlined in Figure S3). Flow cytometry was used to confirm both cell-specific enrichment and viability. As shown in Figures S3B and C, 96.1% of epithelial-enriched cells were EpCAM+ (with a viability of 60%), 91.2% of macrophage-enriched cells were F4/80+ (with a viability of 82.4%), and 97.7% of fibroblast-enriched cells were CD45-/CD90.2+ (with a viability of 78%). Both cell numbers and thus recovery was substantially greater in mammary tumors when compared to mammary glands.

**Fig. 5:**
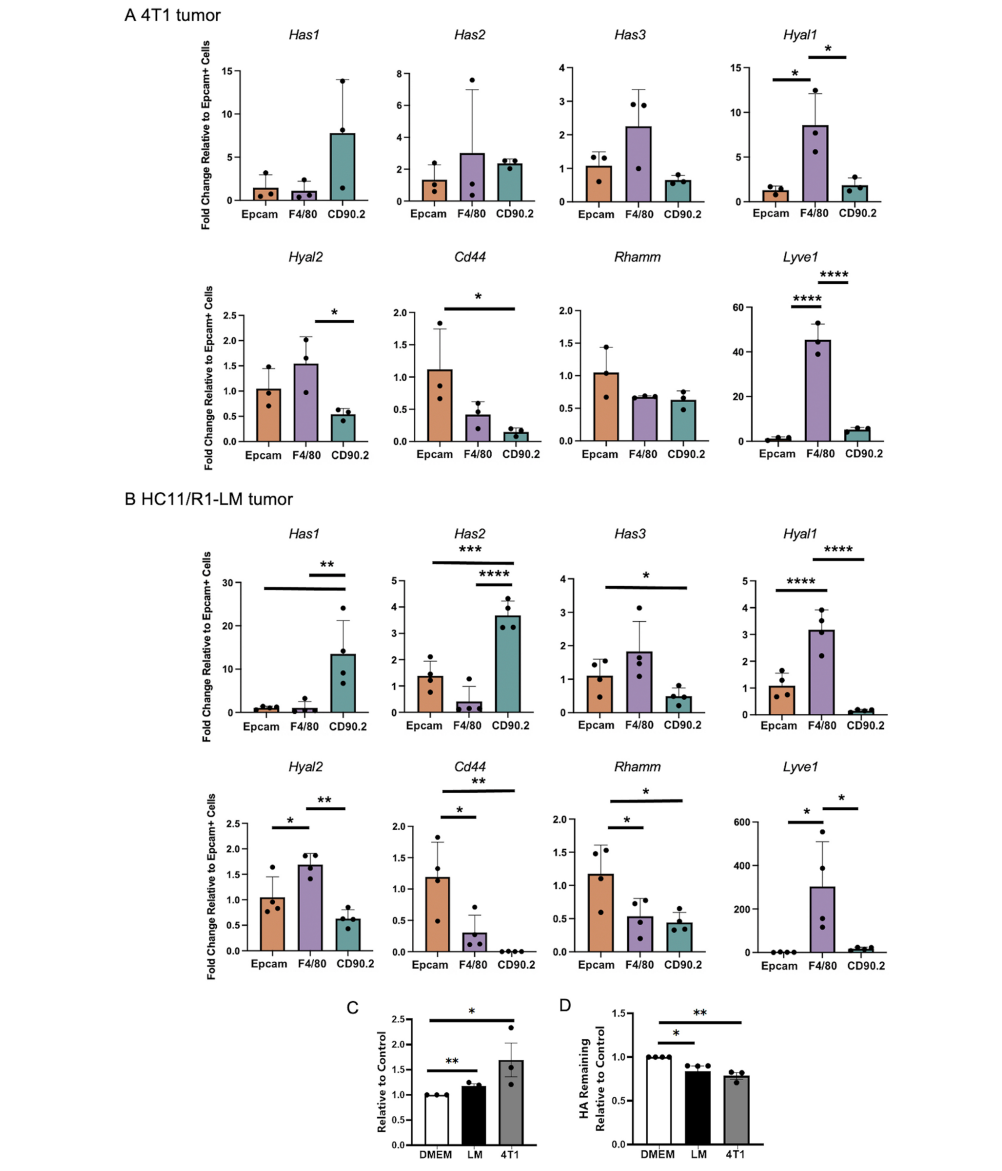
**Contributions to HA synthesis and turnover by cell type in mammary tumors.** EpCAM+, F4/80+, and CD45-/CD90.2 + cells were isolated from (**A**) 4T1 (n = 3) and (**B**) HC11/R1-LM (n = 4) tumors using the Miltenyi Bead Isolation kit. Gene expression by qRT-PCR was analyzed utilizing a panel of HA-related genes: hyaluronan synthesis (*Has1-3*), hyaluronidases (*Hyal1,2*), and hyaluronan receptors (*Cd44, Rhamm, Lyve1*). (**C**) J774 macrophages were incubated with DMEM, HC-11/R1-LM conditioned media, or 4T1 conditioned media and lysates were assayed for hyaluronidase expression by ELISA or **(D)** HA activity with an HA Activity ELISA. Error bars represent standard error of the mean. *P* values * *p* < 0.05; ** *p* < 0.01; *** *p* < 0.001

To investigate HA machinery across mammary tumors, cell-specific gene expression was analyzed within 4T1 (Fig. 5 A) and HC11/R1-LM (Fig. 5B) tumors utilizing a panel of HA-related genes: HA synthesis (*Has1-3*), HA fragmentation (*Hyal1,2*), and HA receptors (*Cd44, Rhamm, Lyve1*). As shown in Fig. 5, EpCAM + cells derived from the 4T1 and HC11/R1-LM tumors had increased expression of *Cd44* compared to the other cell types. This has been shown previously by others, including Miletti-Gonzalez et al. where CD44 was found to be widely expressed on the tumor cells of breast carcinomas [45], thus confirming our findings. EpCAM + cells also expressed elevated levels of *Rhamm* in the HC11/R1-LM tumors.

Perhaps most noteworthy, F4/80 + cells derived from both tumor models expressed elevated levels of *Hyal1*. Because this phenomenon was not observed in normal mammary glands, it suggests that *Hyal1* expression is selectively expressed by TAMs. Furthermore, F4/80 + cells had significantly elevated levels of *Hyal2* expression and robust expression of the HA receptor, *Lyve1*. These data suggest that TAMs play an important role in HA fragmentation in mammary tumors, which has negative prognostic implications for breast cancer patients [33].

Finally, CD45-/CD90.2 + had increased expression of *Has1* and *Has2* when compared to the other cell types, and this was most notable in the HC11/R1- LM tumors. Similar to normal mammary glands, CD45-/CD90.2 + fibroblasts specifically expressed *Has1* whereas EpCAM + epithelial cells expressed elevated levels of *Has3* in the HC11/R1-LM tumor model. This pattern trended in F4/80 + macrophages, although these data were variable and thus not significant.

In summary, these data suggest that cancer cells express elevated levels of the HA receptor, *Cd44*, which may be essential for sensing the matrix-rich environment [46], tumor cell motility/invasion [47], survival, and downstream signaling events [48–50]. CAFs, on the other hand, likely contribute to HA production while TAMs modulate HA fragmentation, both of which have been linked to poor patient survival [17], [33].

### Exposure of macrophages to tumor cell-derived factors increases hyaluronidase expression and activity

Because TAMs derived from 4T1 and HC-11/R1-LM tumors exhibit increased *Hyal* gene expression compared to epithelial cells and fibroblasts (Fig. 5 A,B), we sought to determine whether tumor cells are capable of inducing HYAL protein expression and activity in macrophages. J774 macrophages were stimulated with conditioned media collected from 4T1 and HC-11/R1-LM cells, and HYAL expression and activity were assessed by a pan-HYAL ELISA. As shown in Fig. 5 C, exposure of J774 cells to conditioned media from both tumor cell lines led to increased HYAL protein expression compared to the DMEM control (Fig. 5 C). J774 lysates were assayed for functional HYAL activity and incubated in a 96-well plate precoated with 5 ug of HA. HYAL activity is measured by quantifying the amount of HA remaining in each well. J774 macrophages exposed to HC-11/R1-LM and 4T1 conditioned media fragmented more HA compared to the DMEM control (Fig. 5D). Therefore, macrophages exposed to tumor cell derived factors demonstrate an increased functional ability to fragment HA.

## Discussion

The ECM is biochemically and biomechanically important for mammary gland development [18–22]. Apart from a few key studies [31], [32], HA metabolism has not been investigated in the normal mammary gland literature. HA homeostasis requires a well-balanced, tightly controlled system under physiologic conditions; however, this system is often hijacked in cancer [9], [10]. While there is a growing body of correlative evidence [11], [13–17], the causal relationship between stromal/tumor cells and ECM manipulation in breast cancer development is poorly understood. Therefore, additional work is needed to better understand HA regulation in mammary gland development and subsequent dysregulation in mammary tumorigenesis.

To our knowledge, we are the first to characterize HA deposition within the murine mammary gland across key stages of puberty, pregnancy, and involution. Furthermore, our results suggest that fibroblasts (via *Has1* and *Has2*), epithelial cells (via *Has3*) and macrophages (via *Has3*) contribute to HA synthesis, while macrophages contribute to HA degradation (via *Hyal2*) in the pubertal mammary gland, leading to an organized assembly of HA. Unfortunately, those processes required for ductal morphogenesis are likely advantageous for breast cancer progression [51], [52]. Specifically, our findings suggest a novel role for TAMs in the direct degradation of HA in the TME (via *Hyal1*, *Hyal2*, and *Lyve1*).

HAS2 is vital for embryonically developing tissues, including cardiovascular [3], spinal [53], and craniofacial development [54]. HA synthesis drives mesenchymal cell migration and hydraulically expands tissues, allowing cells to reach their programmed destination [55]. The mammary gland is unique in that the majority of development occurs after birth during puberty, pregnancy/lactation, and involution [18], [20]. In order to understand HA in mammary gland development, a key step is to characterize its localization pattern. We discovered that HA extends throughout the mammary fat pad in an organized fashion, dividing the gland into distinct regions via HA-rich septae and encasing it in a HA-rich fibrous capsule. Although key structures did not significantly change throughout development, we often found epithelial ducts located along, and nestled within HA-rich septa. Our findings are supported by Tolg and colleagues [31] in which HA was important for ductal epithelial-cell branching via EGF signaling in vitro using a 3D culture system. Our data add to this growing body of evidence suggesting that HA is an important matrix component within the developing mammary gland.

We next wanted to investigate expression levels of HA machinery in key cell types within the mammary gland. To characterize cell-specific HA machinery, we isolated EpCAM + epithelial cells, F4/80 + macrophages, and CD45-/CD90.2 + fibroblasts from the mammary glands of 5- (puberty) and 10-week (adult) old mice. Interestingly, *Cd44* expression was elevated in epithelial cells, which may assist in epithelial adhesion and migration during ductal morphogenesis [56]. Others have also shown that CD44 is important for tubule and branch formation in renal epithelia [57] and mammary cells [58], respectively. Therefore, CD44 expression on mammary epithelial cells may be important for epithelial sensing of the matrix-rich environment [46], supporting epithelial cell migration [47] and cell survival [48–50] throughout ductal elongation and branching.

Dermal fibroblasts are known producers of HA [42]. Likewise, we found that fibroblasts derived from developing mammary glands have upregulated expression of *Has1* and *Has2* at both 5- and 10- week time points. Interestingly, *Has1* expression was specific to fibroblasts – this has been observed previously in human skin fibroblasts at both the basal and growth-factor stimulated levels [59]; however this appears context dependent as human lung fibroblasts were shown to only express both *Has2* and *Has3* [60]. Finally, it is important to note that both macrophages and epithelial cells expressed elevated levels of *Has3* when compared to fibroblasts. These gene expression data suggest that all three cell types contribute to HA synthesis within the developing mammary gland. However, future studies are needed to validate cell specific changes at the protein level.

We have shown previously that in vivo depletion of resident macrophages leads to a dramatic increase in both collagen and HA within the mammary gland stroma of nulliparous mice [32]. In the current studies, we found that *Hyal2* expression was significantly upregulated in macrophages when compared to fibroblasts at the 5-week time point, further emphasizing a role for macrophages and HA turnover in pubertal mammary glands. Collectively, these data are supported by a recent finding in which LMW HA fragments (7–21 kDa) were isolated from the mammary gland during pregnancy [31]; however, this study did not investigate HA fragmentation during puberty. Therefore, additional studies are needed to define macrophage-specific HA regulation throughout pregnancy, lactation, and involution. Moreover, future work is needed to identify nuances in subpopulations. For example, we have previously identified a specific subpopulation of LYVE1 + macrophages expressing a variety of ECM remodeling genes at higher levels than LYVE1- macrophages [32]. Therefore, our current studies may not fully capture the complex roles of macrophages in matrix homeostasis due to our bulk-analysis approach.

By understanding HA regulation in wildtype mammary glands, it is logical to next infer how cancer cells might exploit these “normal” roles for tumorigenesis. We began by characterizing HA deposition within two murine models of breast cancer: 4T1 and HC11/R1-LM. In contrast to the organized HA-rich septa found within normal mammary glands, HA was chaotically deposited throughout mammary tumors, a pattern which has previously been linked to poor patient survival [17], [43]. While these studies were performed in two aggressive orthotopic injection-based murine models of breast cancer (4T1 and HC11/R1-LM), previous work in our lab demonstrates increased HA deposition in the mammary gland in an autochthonous model of tumorigenesis [61]. However, additional work is needed to fully characterize changes in HA deposition throughout breast cancer progression. Specifically, comparing HA deposition and organization across various breast cancer models, including autochthonous and orthotopically transplanted tumor models, will provide additional insights into the importance of HA organization in breast cancer.

To investigate which cell-types contribute to aberrant regulation of HA synthesis and turnover, EpCAM + epithelial cells, F4/80 + macrophages, and CD45-/CD90.2 + fibroblasts were isolated from 4T1 and HC11/R1-LM mammary tumors. Comparable to normal mammary glands, CD45-/CD90.2 + fibroblasts had increased expression of *Has1* and *Has2* when compared to the other cell types, which was most notable in the HC11/R1-LM tumors. Thus, our data support previously published work suggesting that CAFs contribute to HA production within the TME [62–64]. Importantly, Brichkina et al. demonstrated that CAF-mediated HA synthesis via HAS2 promoted lung cancer growth in vitro [64].

Additionally, we found elevated *Cd44* expression in tumor cells derived from the aggressive 4T1 and HC11/R1-LM models of breast cancer. These findings are consistent with others in which CD44 expression was upregulated in breast carcinomas [48], [65], [66]. Upregulation of CD44 is likely associated with aggressive disease since CD44 is important for matrix binding/motility [47] (leading to MMP expression and thus ECM degradation [67]), cell aggregation and subsequent metastasis [66], and cell survival [48–50].

Strikingly, F4/80 + macrophages derived from both tumor models expressed elevated levels of *Hyal1* and *Hyal2*, suggesting that TAMs play a pivotal role in HA degradation. To our knowledge, this is the first piece of evidence linking TAMs to the direct degradation of HA within the TME of mammary tumors. This knowledge is powerful, as LMW HA binding to CD44 has been shown to promote cancer-associated inflammation [48], [68]. In addition, we found in vitro tumor cell conditioned macrophages have increased hyaluronidase protein expression and an increased ability to fragment HA. This finding suggests tumor derived factors are driving expression of hyaluronidases in macrophages. Our findings also support others in which tissue reparative, M2-like macrophages correlated with HA accumulation at the invasive front of breast carcinomas [69], [70]. Furthermore, F4/80 + cells had robust expression of the HA receptor, *Lyve1*, in the aggressive tumor models. While LYVE1 + macrophages are important for the maintenance of lymphatic vessels under normal conditions [71], they have recently been linked to ovarian cancer metastasis [72]. Additionally, our previous work identified this unique macrophage subpopulation within the peritumoral stroma of 4T1 mammary tumors [32]. Collectively, the current studies align with existing evidence suggesting that LYVE1 + macrophages are responsible for HA remodeling in breast cancer. Future studies are needed to determine the effects of LYVE1 + macrophage depletion on mammary tumorigenesis in vivo.

Our findings suggest that macrophages play a role in HA turnover in the developing mammary gland, and this role is exploited in TAMs. Since HA degradation has been shown to foster breast cancer progression, manipulation of macrophage polarization or inhibition of TAM-mediated HA-degradation may be an exciting new approach for the treatment of breast cancer.

## Electronic Supplementary Material

Below is the link to the electronic supplementary material.

Supplementary Material 1
