# Supplementary material for: Characterization of Hyaluronan Localization in the Developing Mammary Gland and Mammary Tumors
Source: J Mammary Gland Biol Neoplasia. 2023 Jan 27;28(1):1. doi: 10.1007/s10911-023-09528-y (PMC9892096; doi:10.1007/s10911-023-09528-y)
Supplement: Supplementary file 1 — Supplementary Material 1 [file 10911_2023_9528_MOESM1_ESM.pdf]

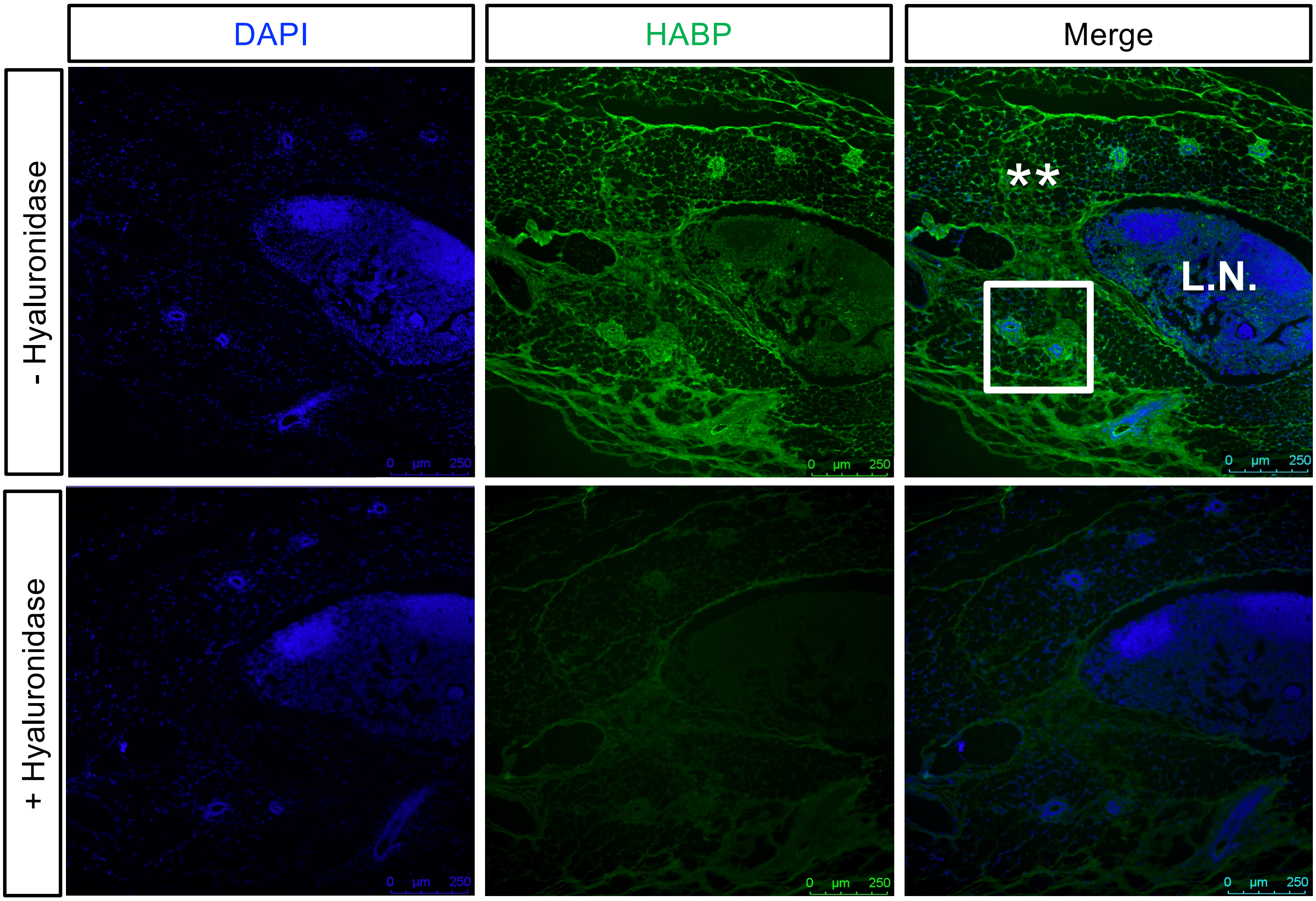

Figure S1. Localization of HA on paraffin-embedded tissues. Hyaluronan (HA) is digested on control slides using hyaluronidase. Both control and experimental slides are treated with biotinylated hyaluronan-binding protein (HABP) followed by secondary streptavidin fluorophore. HA staining is visualized under a fluorescent microscope. Representative images show immunofluorescence microscopy for hyaluronic acid binding protein (HABP; green) and DAPI nuclear stain in a 5-week old (pubertal) murine mammary gland. As a control, each section was treated with hyaluronidase prior to staining (+Hyaluronidase). L.N. represents the lymph node, ** highlights the adipose-rich stroma, and the white box identifies two mammary epithelial buds. Each image was taken at 10× magnification. Images were acquired using the same settings (including exposure time and gain) and post-processing adjustments (including brightness and contrast). Scale bars represent 250 μm.

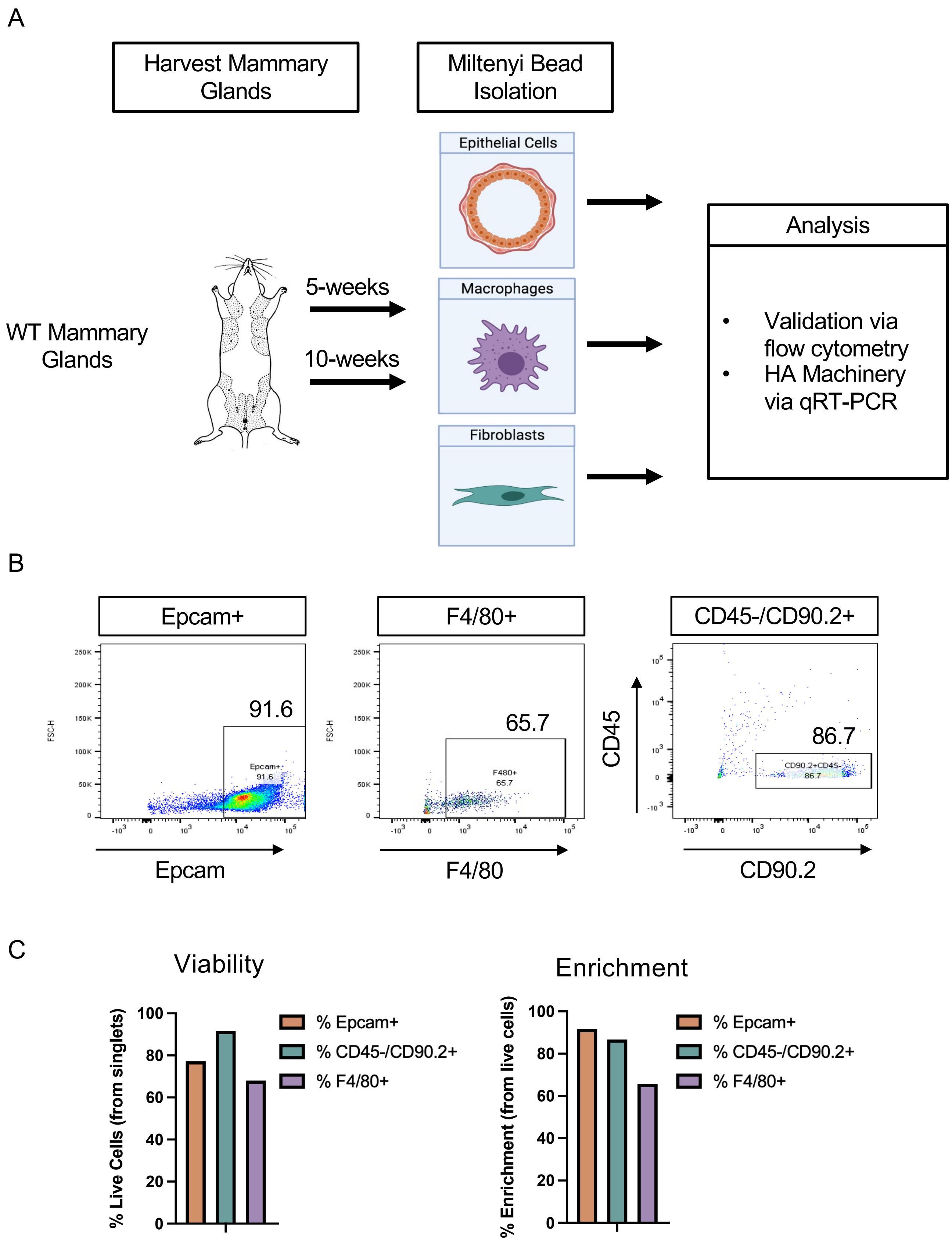

Figure S2. (**A**) Cell-specific isolation of EpCAM+ epithelial cells, CD45-/CD90.2+ fibroblasts, and F4/80+ macrophages from the mammary glands of 5- (pubertal) and 10- (adult) week old BALB/c mice via Miltenyi Biotec microbead kits. Image created with BioRender.com. (**B**) Gating strategy for flow cytometry was analyzed and (**C**) quantified via FlowJo, validating cell viability and enrichment.

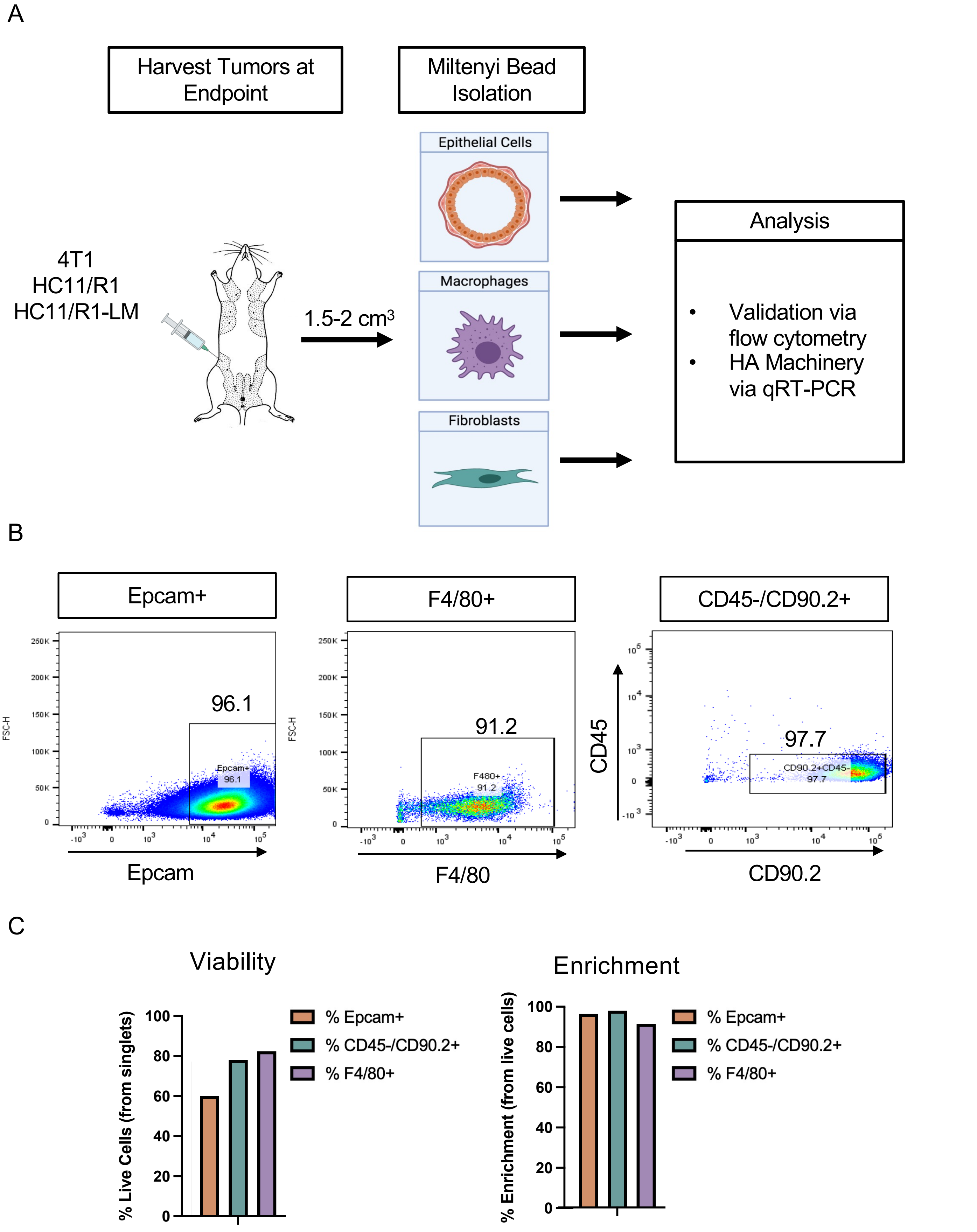

Figure S3. (**A**) Cell-specific isolation of EpCAM+ epithelial cells, CD45-/CD90.2+ fibroblasts, and F4/80+ macrophages from three murine models of breast cancer (4T1, HC11/R1, and HC11/R1-LM) via Miltenyi Biotec microbead kits. Image created with BioRender.com. (**B**) Gating strategy for flow cytometry was analyzed and (**C**) quantified via FlowJo, validating cell viability and enrichment.
